# Synthetic data in medical imaging within the EHDS: a path forward for ethics, regulation, and standards

**DOI:** 10.3389/fdgth.2025.1620270

**Published:** 2025-10-08

**Authors:** Junying Jiang, Lúcia Domingues, Jorge M. Mendes

**Affiliations:** Comprehensive Health Research Centre (CHRC), NOVA Medical School, Faculdade de Ciências Médicas, Universidade NOVA de Lisboa, Lisbon, Portugal

**Keywords:** synthetic data, medical imaging, European Health Data Space (EHDS), data privacy, generative AI, GDPR compliance, AI act, Medical Device Regulation (MDR)

## Abstract

The increasing availability of medical imaging data offers unprecedented opportunities for advancing artificial intelligence (AI)-driven healthcare. However, strict data protection regulations in the European Union (EU), especially the General Data Protection Regulation (GDPR), present significant challenges to data sharing and reuse. Synthetic data—artificially generated data that mimic the statistical properties of real data without revealing sensitive information—have emerged as a promising solution to bridge this gap. This perspective-style review examines the role of synthetic medical imaging data within the European Health Data Space (EHDS), a policy initiative aimed at enabling secure access to health data across the EU. While we briefly reference cross-cutting privacy-enhancing technologies and one non-imaging comparator to illuminate shared governance issues, our analysis and conclusions are scoped to imaging applications. We discuss the technical foundations and types of synthetic data, their potential to enhance reproducibility and innovation, and the complex ethical and legal concerns surrounding their use. Emphasising the need for a risk-based regulatory framework, we advocate for synthetic data governance that ensures utility, transparency, and accountability, especially when such data are generated using generative AI models. This work contributes to ongoing debates on how synthetic imaging data can support a privacy-preserving, data-driven healthcare ecosystem in Europe.

## Introduction

The European Health Data Space (EHDS) is a significant initiative that facilitates cross-border data sharing within the European Union (EU). EHDS aims to advance healthcare research, artificial intelligence (AI) development, and patient care by enabling access to healthcare data. Medical imaging data, which are crucial for diagnostics and treatment planning, encounter substantial obstacles to sharing owing to stringent privacy laws, ethical concerns, and security challenges. These barriers pose significant challenges to the development and deployment of innovative AI solutions in healthcare. Synthetic data that replicate realistic imaging datasets without compromising patient privacy have emerged as a promising solution to these challenges, particularly in the early stages of clinical validation. This review examined the potential of synthetic data in medical imaging, focusing on its alignment with EHDS objectives, ethical considerations, regulatory compliance, and the establishment of industry standards. Specifically, it aims to (1) define synthetic data and its applications in medical imaging within the EHDS framework; (2) demonstrate how synthetic data can address barriers to clinical validation, particularly in the early stages of innovative solution development; (3) explore the ethical and regulatory considerations related to the use of synthetic imaging in the development and clinical validation; and (4) propose best practices to ensure synthetic data quality, interoperability, and applicability to support real-world clinical use cases. Our focus is **synthetic medical imaging** in the EHDS context. We occasionally draw on cross-cutting PETs and one contextual (non-imaging) comparator solely to clarify the same regulatory and ethical questions (e.g., Recital 26 anonymity, controller/processor roles, AI Act risk). Unless explicitly noted, all claims, recommendations, and the governance toolkit are intended for imaging.

## Methodology

This literature review employed an established and transparent framework to identify and synthesise relevant literature on the ethical and regulatory considerations of using synthetic medical image data within the EHDS context. Although the structure of this review draws inspiration from established frameworks, it does not fully conform to the PRISMA (Preferred Reporting Items for Systematic Reviews and Meta-Analyses) guidelines.

To ensure broad coverage and relevance, a structured search was conducted across four major databases: PubMed, Scopus, Web of Science, and Google Scholar. The initial search was carried out on December 21st, 2024, with an update on January 5th, 2025 to capture any newly added studies. Google Scholar was searched last to capture potential grey literature. In addition to database searches, forward and backward citation tracking was performed by screening the reference lists of included studies to identify further relevant works (leading to the addition of **29** studies).

Search terms were developed to reflect the core themes of the review in consultation with subject-matter experts. Keywords included combinations of terms related to synthetic data, medical images, EHDS, ethics, applications, and clinical relevance. Consistent terms were used across all databases except PubMed, where *Medical Subject Headings* (MeSH) were applied as appropriate.

The review focused on studies addressing ethical and regulatory issues related to synthetic medical image data within the EHDS framework. The inclusion criteria were:

• English-language publications.
• Published between January 1st, 2020 and January 5th 2025.
• Studies discussing synthetic data, especially in medical imaging, with a link to legal, ethical, or clinical applications.

**Figure 1:**
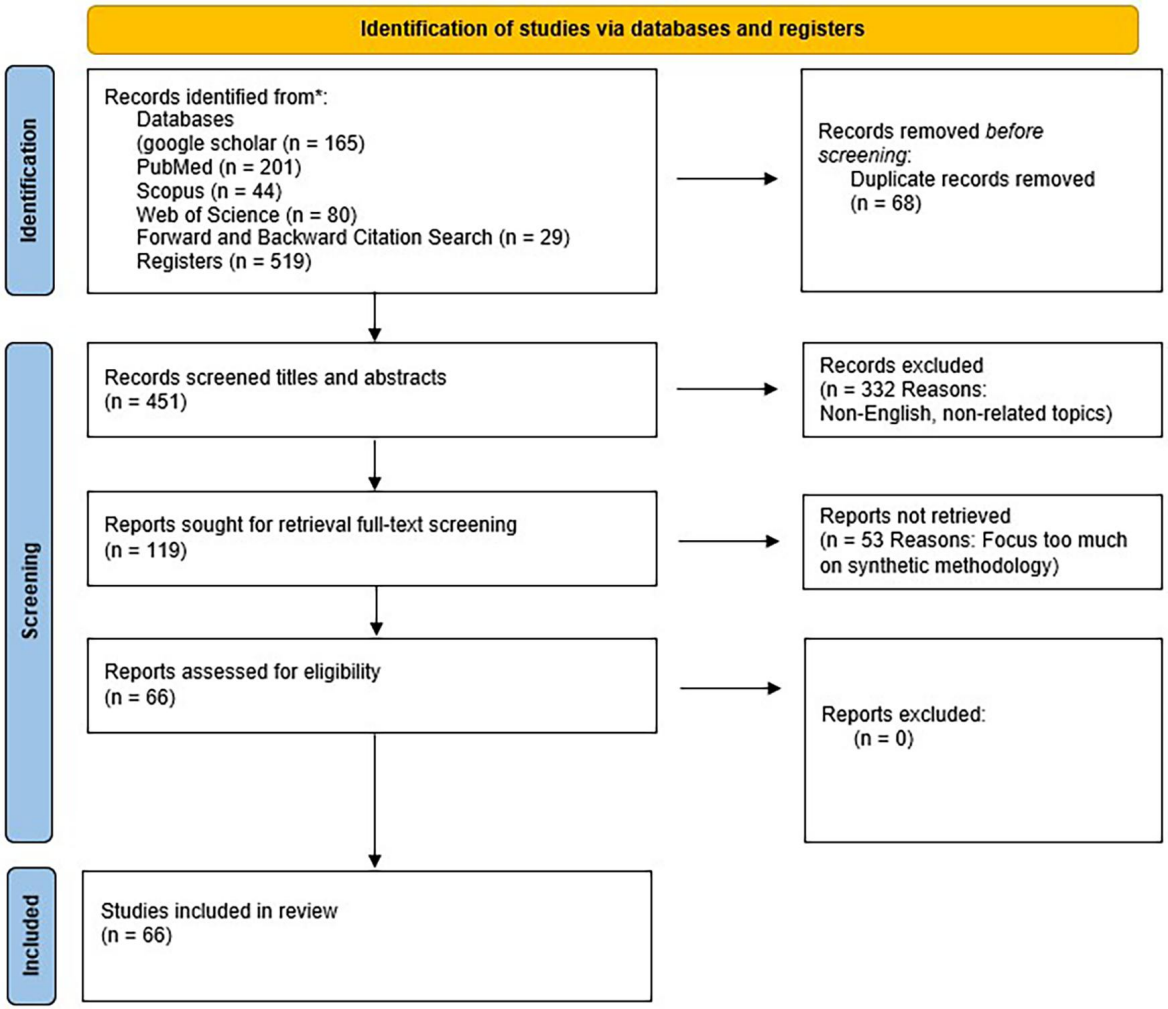
Flowchart representing of the selection of the included studies.

Given the broad scope of the topic, the search also employed multiple subsets of keywords to maximise sensitivity and relevance. From an initial pool of **516** studies, **68** duplicates were removed. Titles and abstracts of the remaining studies were screened against pre-defined inclusion criteria, excluding **332** papers. The full texts of **119** remaining articles were then assessed, of which **53** were excluded for not meeting the eligibility criteria. A total of **66** studies were ultimately included in the final analysis. These were included because relatively few publications addressed the four core objectives of this review in an integrated manner. The complete screening results are summarised in Table 1, and the study selection process is illustrated in Figure 1.

**Table 1:** Screening results.

Database	Search results	Search results	Results	Results after
	(December 21st, 2024)	update (January 5th, 2025)	(duplicated removed)	screening
Google scholar	165	165	165	37
PubMed	163	201	144	
Scopus	44	44	37	
Web of science	76	80	76	29
Forward and backward				
Citation search	29	29	29	
Total	477	519	451	66

This literature review adopted a structured and rigorous process to identify and analyse studies relevant to using synthetic medical image data ethically within the EHDS framework. Although the review was not formally registered or reported according to PRISMA standards, key elements—such as transparent inclusion criteria, structured searching, and screening by multiple reviewers—were incorporated to enhance robustness.

Given the limited literature directly addressing synthetic image data under EHDS, the included studies were organised into thematic subtopics:

• Introduction to synthetic data;
• Synthetic data within EHDS or related legal/ethical frameworks;
• Applications in medical imaging;
• Implementation and technical considerations.

Each full-text article was assessed independently by the authors. Any disagreements during screening or categorisation were resolved through discussion. Relevant content was highlighted and categorised for use in the manuscript. This structured methodology enabled the development of a comprehensive and credible synthesis, supporting a nuanced discussion of generalisability, ethical compliance, and practical implementation of synthetic image data in the European health research and innovation landscape.

We scoped this review to ethical and regulatory aspects of synthetic medical imaging under the EHDS. A small number of contextual (non-imaging) sources are cited where they inform the same governance questions (e.g., GDPR anonymisation tests, secondary-use access via data access bodies, and high-risk AI obligations).

## Synthetic data and its role in EHDS

With an imaging focus, we discuss how the EHDS is designed to enhance the sharing and reuse of health data across the EU. Where we reference LLMs/EHR or non-imaging PETs, these are used as cross-cutting comparators to the same governance issues faced by imaging. The conclusions remain imaging-scoped unless explicitly stated. EHDS establishes a health-specific ecosystem for primary and secondary health data use (1–6). By clarifying the scope of secondary data use, the EHDS aims to facilitate the secure exchange and interoperability of health data throughout Europe (1).

EHDS addresses the challenges associated with increasing the volume and complexity of health data. These challenges include data bottlenecks resulting from the sensitivity of health information, interoperability issues due to a lack of standardisation, and fragmentation, which complicate the navigation of health services and data protection frameworks (6). To mitigate these issues, EHDS proposes a unified market for electronic health record systems, relevant medical devices, and high-risk AI-driven healthcare applications. It establishes governance frameworks, common standards, and infrastructure to ensure seamless data exchange from decentralised sources (1, 6).

A fundamental aspect of EHDS is the assurance that secondary use of data complies with ethical and legal standards, particularly in relation to the General Data Protection Regulation (GDPR). The EHDS enhances data access rights, establishes governance structures at the Member State level, and requires standardised formats for health data systems (1). Nonetheless, a significant challenge persists: facilitating extensive data sharing, while safeguarding patient privacy and mitigating the risks associated with data breaches.

Synthetic data represent a promising solution within the EHDS framework, enabling stakeholders to utilise health data for research, AI model training, and policymaking without compromising patient confidentiality (4). Synthetic data consist of artificially generated datasets that maintain the statistical properties of real-world health data while removing personally identifiable information. This approach aligns with the objective of maximising the utilisation of health information, while ensuring legal compliance (4). Medical images, for instance, fall under the broader category of health data that the EHDS aims to make interoperable and accessible through the digitisation of health records across the EU (7). Synthetic medical imaging data enables researchers and clinicians to develop and test AI modelsat the early stages without accessing sensitive patient information.

Although a legal definition of synthetic data is absent, it is commonly recognised through its generation methodologies. Ianese (4) characterises synthetic data as artificial information derived from real datasets using AI techniques. Despite its fictitious nature, synthetic data retains the statistical properties of the original dataset, rendering it valuable for the research and training of machine learning models. Similarly, Greser (8) contends that synthetic data are not collected, but are instead generated algorithmically to mirror the statistical distributions of real-world data, with its utility contingent upon the accuracy and representativeness of the original dataset.

Various techniques have been used to generate synthetic health data. Generative Adversarial Networks (GAN) consist of a generator and discriminator, which collaboratively generate synthetic samples that closely mimic real data. Conditional GAN (CGAN) enhance this framework by integrating additional information, whereas Conditional Tabular GAN (CTGAN) further refine CGAN for structured health data by addressing rare occurrences through mode-specific normalisation (9). Autoencoders are neural networks that compress the input data into a latent representation before reconstructing them. Variational Autoencoders (VAE) advance this approach by ensuring that the latent space adheres to a Gaussian distribution (9). Alternative methodologies include *SynthPop*, which generates synthetic data sequentially based on conditional distributions; *Maximum Spanning Tree* (MST) methods, which preserve noisy marginals of low-dimensional data distributions, and *PrivBayes*, which constructs Bayesian networks and introduces noise into marginal statistics (9).

Synthetic data can be generated from actual electronic medical records, preserving their statistical properties, while ensuring the exclusion of any real patient data. In contrast to anonymisation techniques, synthetic data retains the essential characteristics of the original dataset while adhering to privacy standards (4).

The increasing deployment of Large Language Models (LLM) in the healthcare sector presents significant challenges related to data availability and quality. Access to high-quality medical data is frequently restricted by paywalls and regulatory constraints, thereby limiting the training of domain-specific AI models. Synthetic data have emerged as a viable solution, as they allow for the expansion of dataset size while ensuring privacy (10). A recent study introduced a medical LLM, *GatorTronGPT*, which was developed using synthetic data, resulting in enhanced clinical text generation performance compared with models trained solely on real data (11). Consequently, synthetic data generation effectively addresses data scarcity, enhances model performance, and improves generalisation, while safeguarding privacy.

The SECURED (Scaling up Secure Processing, Anonymisation and Generation of Health Data for EU Cross-Border Collaborative Research and Innovation) project, launched in January 2023, aims to enhance multiparty computation, data anonymisation, and synthetic data generation within the health sector. A significant application of this project is the creation of synthetic data for educational purposes, enabling medical students to engage with realistic case studies without compromising actual patient records (12) while ensuring privacy in cross-border data sharing (13).

Research in medical imaging has benefited significantly from the utilisation of synthetic data. Traditional datasets in medical imaging frequently encounter limitations such as insufficient sample sizes, data heterogeneity, and challenges in integration owing to variations in imaging protocols across different institutions. Synthetic medical imaging data can effectively address these issues by generating diverse and high-quality training sets, thereby enhancing deep learning models for diagnostic and treatment planning purposes (10).

Although the integration of synthetic data into the EHDS framework offers numerous benefits, it also presents significant ethical and regulatory challenges. The absence of standardised quality metrics and regulatory classification under the GDPR raises concerns regarding data bias and validity (14, 15). Nevertheless, advancements in deep generative models and privacy-preserving infrastructure, such as EHDS health data nodes, provide potential solutions to these issues. Ensuring transparency in the data generation and validation processes is crucial for maximising the potential of synthetic data while adhering to ethical and legal standards.

## Ethical challenges of synthetic data

While synthetic data is increasingly promoted as a privacy-preserving alternative to real-world health data, its use in research and development of innovative health solutions introduces complex ethical considerations beyond data protection compliance. Central among these are concerns related to consent, re-identification risks, bias, scientific integrity, data sovereignty, and cybersecurity (8, 9, 16–19). These issues challenge the assumption that synthetic data is inherently free from ethical scrutiny and underscore the importance of governance frameworks that align with legal requirements and ethical research principles.

### Informed consent and data subject autonomy

Although synthetic data does not directly contain identifiable personal information, it is typically generated from real datasets originally contributed by individuals. In many cases, these individuals may not have explicitly consented to the use of their data for synthetic data generation or secondary purposes such as model training, commercial use, or cross-border data sharing (19, 20). This raises important ethical concerns regarding autonomy, transparency, and respect for data subjects’ rights—particularly when synthetic data is used beyond the original purpose for which the real data were collected (21). Ethical research demands that participants be properly informed about how their data may be used, even in derived or non-identifiable forms. This is in line with the 2024 revision of the Declaration of Helsinki, which emphasizes that researchers must obtain free and informed consent for the collection, use, storage, and possible future use of biological materials and identifiable or re-identifiable data. The Declaration also states that if future uses cannot be fully foreseen at the time of consent, they must still be approved by an ethics committee, especially when obtaining new consent is not practical. These updates reflect the growing importance of transparency and ethical oversight in data reuse and secondary research. The European Data Protection Supervisor (EDPS) has emphasised that scientific research cannot serve as a “carte blanche to take irresponsible risks” and must be conducted within an established ethical framework (22).

In response to the tension between strict consent requirements and the need for flexible data use, concepts such as “broad consent” have emerged. Broad consent allows future research uses while upholding ethical standards through ongoing information, oversight, and participant engagement (23). However, this model remains controversial, particularly when applied to data used to generate synthetic datasets that could be reused in ways not foreseen during initial data collection.

### Privacy risks and re-identification

Although synthetic data is designed to eliminate personally identifiable information, it does not entirely remove the risk of privacy breaches. According to the G29 Working Party and CNIL, there are three key risks associated with synthetic data derived from anonymised sources: (1) Singling Out, where unique data points lead to the identification of individuals; (2) Linkability, where records across datasets can be connected; and (3) Attribute Inference, where sensitive characteristics of individuals may be deduced (9). These risks are especially pronounced in contexts involving small populations or rare diseases, where the uniqueness of cases increases re-identification potential (19).

Generative Adversarial Networks (GANs), commonly used to generate synthetic medical images, can inadvertently reproduce features of the original dataset if not properly regularised. It may result in membership inference attacks, whereby an adversary can determine whether a particular individual’s data was used in the training set (24). For example, in a study involving adolescents with de novo mutations, a GAN trained on facial phenotype data was shown to be vulnerable to such attacks if model weights were publicly shared (24). Differential privacy techniques have shown promise in mitigating these risks (25). Still, they are not foolproof and can introduce trade-offs with data utility.

The legal ambiguity surrounding the classification of synthetic data adds another layer of complexity. Under the GDPR, data is only considered anonymous if re-identification is not reasonably possible, considering time, cost, and technology (23). However, the threshold for what constitutes “reasonable” varies across jurisdictions. Institutions such as biobanks may not hold re-identification keys. However, they could still be subject to data protection regulations if synthetic data is classified as pseudonymised (1).

### Bias and fairness

Synthetic data generation is highly dependent on the quality and diversity of the source data. If the original dataset is unbalanced or reflects systemic inequalities—such as underrepresenting minority populations or biased clinical practices—these issues are replicated or amplified in the synthetic data (6, 18). For instance, chest X-ray datasets have been shown to underrepresent certain demographic groups, leading to reduced diagnostic performance for these populations (26).

While differential privacy techniques are designed to protect sensitive attributes, they may disproportionately affect minority subgroups by introducing statistical noise that distorts already limited representation (19). Several strategies have been proposed to address these challenges, including dataset rebalancing, fairness-aware GANs (such as HealthGAN), adversarial training, and participatory design approaches that involve community stakeholders in model development (14, 27).

### Scientific integrity and trust

A critical concern in the use of synthetic data is the potential erosion of scientific integrity and public trust. Unlike real-world datasets, synthetic data lack an intrinsic link to empirical reality, raising questions about their validity for hypothesis testing, model training, and clinical decision-making. The absence of standardised benchmarks for evaluating synthetic data quality further complicates this issue (10, 19).

Studies have shown that different types of synthetic distribution shifts may not improve model robustness and may even obscure performance limitations under real-world conditions (18). Moreover, the use of synthetic data without full disclosure of generation methods, limitations, and validation procedures can mislead users about the reliability of research findings. Transparent reporting and the development of synthetic data quality standards are essential to ensure credibility in research outcomes (17).

### Data sovereignty and indigenous data ethics

Ethical considerations are particularly salient when synthetic data involves populations with specific historical and cultural vulnerabilities, such as Indigenous communities. Historically, these populations have faced misuse and exploitation of their health data. Using synthetic data does not eliminate the need for appropriate governance; instead, it introduces new complexities related to data sovereignty and community engagement (17).

Excluding Indigenous populations from training datasets may lead to reduced model accuracy for these groups, while including them—without community oversight—may infringe upon their rights to control their data. Synthetic Indigenous datasets must be developed in consultation with the communities they aim to represent, ensuring alignment with ethical principles such as ownership, control, access, and possession (OCAP).

### Cybersecurity and misuse risks

Synthetic data also introduces cybersecurity vulnerabilities that anonymisation may not fully mitigate. Techniques such as data poisoning and adversarial attacks can compromise model integrity at various stages of the AI lifecycle (8). In medical imaging, even minor alterations to input data can cause misclassification, potentially resulting in harmful clinical outcomes.

Partially synthetic datasets combining real and artificial data are particularly susceptible to data leakage and exploitation. Malicious actors may attempt to reverse-engineer synthetic datasets to infer information about real individuals, especially when model architectures and training parameters are publicly available (19).

Synthetic data holds significant promise for enabling innovation while preserving individual privacy. Yet, it is not inherently exempt from ethical scrutiny. Concerns surrounding informed consent, re-identification, bias, scientific reliability, and community engagement highlight the need for robust ethical oversight. Addressing these challenges requires interdisciplinary collaboration, standardised evaluation frameworks, and continuous engagement with stakeholders (28). Only through a deliberate and ethically grounded approach can the benefits of synthetic data be fully realised without compromising individual rights or public trust.

## Regulatory frameworks and standards development

Currently, the European Union lacks dedicated regulations that specifically govern the use of synthetic data. While this absence of direct legislative instruments might suggest legal permissibility, it should not be interpreted as an absence of oversight. Synthetic data—particularly in healthcare and AI applications—may still fall under existing frameworks such as the General Data Protection Regulation (GDPR), the Medical Device Regulation (MDR), and the forthcoming AI Act. These instruments impose obligations concerning data quality, privacy, transparency, and accountability, which are highly relevant when synthetic data is used in developing, validating, or deploying medical AI systems (8). This section examines the regulatory, standard, and framework considerations related to the generation and use of synthetic imaging data, emphasising its transformative potential to surmount traditional barriers in healthcare data sharing. The objective is to present a balanced yet supportive perspective on synthetic data, underscoring its alignment with regulatory frameworks and its capacity to drive equitable healthcare innovation across Europe.

### European regulatory instruments

1. General Data Protection Regulation (GDPR)
  Although synthetic data aims to eliminate identifiable personal information, it may still fall under the scope of the GDPR if the risk of re-identification persists. Article 4(1) defines personal data broadly, and Recital 26 clarifies that data is only anonymous if re-identification is not reasonably possible. Consequently, synthetic data must be evaluated case-by-case to determine whether it meets this threshold. Where it does not, obligations related to lawful processing, purpose limitation, and data minimisation remain applicable (29, 30).
2. European Health Data Space (EHDS)
  The proposed EHDS regulation seeks to establish a harmonised framework for using and reusing health data across the EU. While the regulation prioritises pseudonymised data, it acknowledges anonymised and synthetic data as potential tools for secure secondary use. However, detailed provisions for synthetic data are not yet fully articulated, raising questions about its governance under the EHDS framework (14, 15).
3. AI act
  The proposed AI act introduces a risk-based approach to regulating AI systems, including those used in medical and healthcare contexts. While the Act does not refer explicitly to synthetic data, it encompasses training data quality, robustness, and transparency—directly relevant to synthetic dataset generation. Developers using synthetic data to train or validate AI systems may be required to demonstrate the quality and representativeness of their data, especially in high-risk applications (31).
4. Medical device regulation (MDR)
  Under the MDR, AI systems used for medical purposes may qualify as medical devices, particularly if they support diagnostic or therapeutic decisions. Synthetic data for designing, testing, or validating such systems must be robust, clinically relevant, and traceable. Although the MDR does not yet contain specific provisions for synthetic data, its emphasis on clinical evidence and performance evaluation implies that the origin and quality of training data—including synthetic sources—are subject to scrutiny.
5. Global and comparative contexts
  Globally, the practices surrounding synthetic data intersect with regulatory frameworks such as the U.S. Health Insurance Portability and Accountability Act (HIPAA) and China’s Personal Information Protection Law (PIPL). While HIPAA takes a rule-based approach, the GDPR is principle-based, leading to different interpretations and applications of synthetic data protections. In contrast, the PIPL’s limited data set model permits de-identified data to be used for research without explicit consent, demonstrating a more flexible legal framework (32).
6. Standardisation initiatives
  Standardisation bodies such as ISO, IEC, and CEN are working toward frameworks incorporating synthetic data within broader AI and data governance standards. Emerging standards—such as ISO/IEC 38507 and ISO/IEC TR 24028—provide guidance on the ethical use of AI and risk management, which may be extended to include the generation and validation of synthetic data. The European Commission has played a central role in supporting these efforts by collaborating with CE and CENELEC to develop harmonised standards for AI data quality under the ISO 5259 series (Burden and Stenberg, 2024). The FUTURE-AI initiative also outlines criteria for trustworthy AI and ethical synthetic data use in medical imaging, focusing on fairness, robustness, and explainability (33). Meanwhile, the FAIR (Findable, Accessible, Interoperable, Reusable) and CARE (Collective Benefit, Authority to Control, Responsibility, Ethics) principles offer valuable guidance on ethical and responsible synthetic data practices, particularly in open science and Indigenous contexts (17, 34). Standardisation is also central to tackling data fragmentation, especially in highly sensitive areas such as paediatric oncology. Tozzi et al. (35), in a systematic review of AI in paediatric brain tumour research, underscores the urgent need for harmonised and interoperable data sources across European institutions to improve reproducibility and model performance.
  Complementary to these efforts, the Fast Healthcare Interoperability Resources (FHIR) standard is key in integrating synthetic datasets into real-world health systems. As discussed by Pereira et al. (10), FHIR defines the structure and semantics of electronic health data, enabling consistent exchange and supporting synthetic data applications through better interoperability, standardisation, and integration across diverse platforms.

### Mapping use cases to regulatory and standards obligations

**Figure 2:**
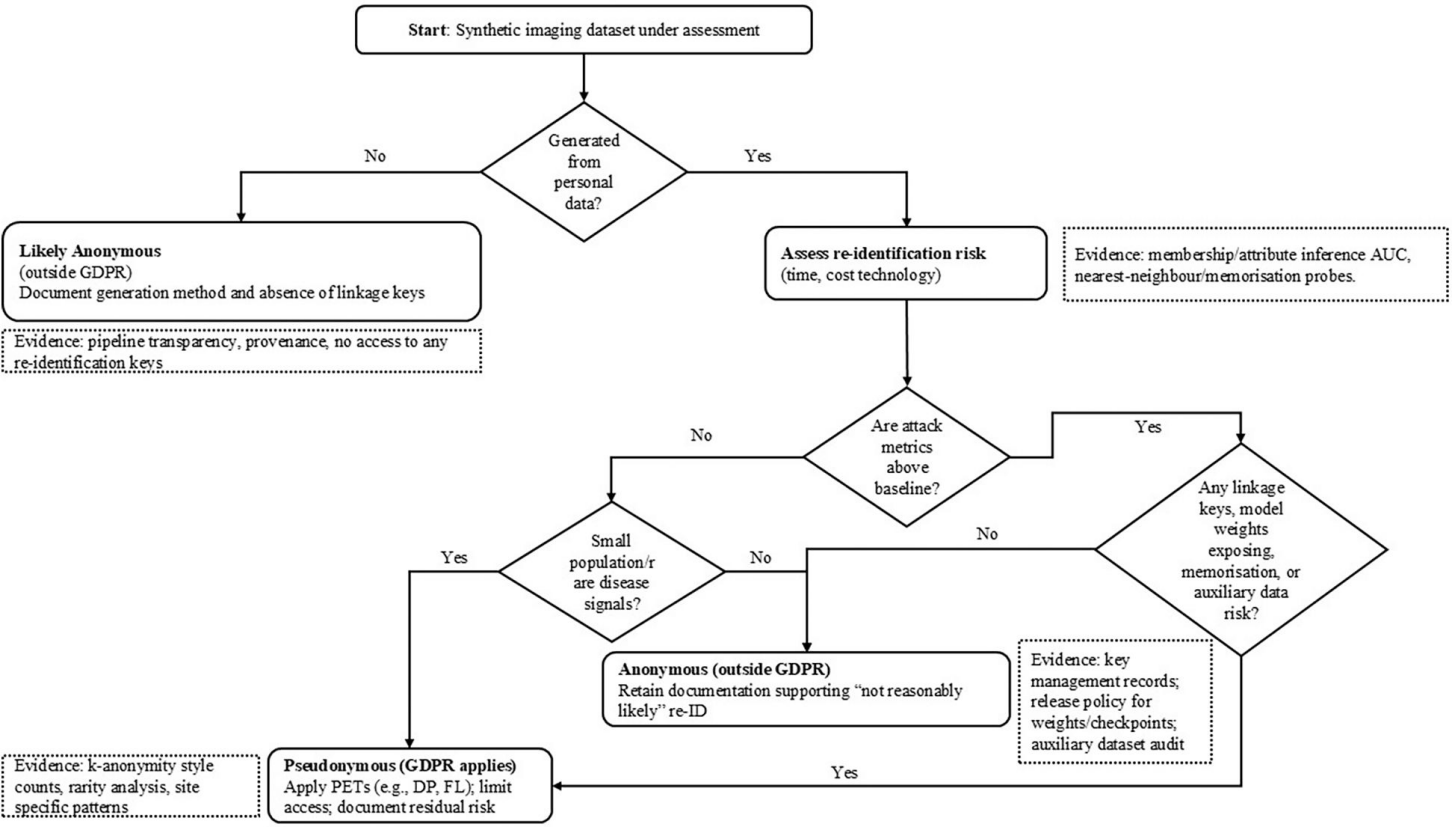
Recital 26 decision tree: is a synthetic imaging dataset anonymous or pseudonymous? Evidence expectations shown in dashed notes.

To translate the preceding legal analysis into practice, Table 2 maps common use cases of synthetic imaging data to obligations under the EHDS, GDPR, AI Act, and MDR. Figure 2 provides a Recital 26 decision tree to assess whether a dataset is anonymous or pseudonymous, together with evidence expectations. Unless explicitly noted as cross-cutting, the following mapping is scoped to synthetic medical imaging.

**Table 2:** Mapping synthetic-imaging use cases to EHDS/GDPR/AI Act/MDR obligations.

Use case	EHDS role${}^{\text{a}}$	GDPR status${}^{\text{b}}$	AI Act risk${}^{\text{c}}$	MDR implications (if clinical)${}^{\text{d}}$
U1. Method development/education (non-clinical)	Controller: research org./data holder defining purpose. Processor: hosting/cloud provider or lab IT.	Often *anonymous* if: no linkage keys retained; MIA/AIA≤baseline; no small-population leak risk; no model weights exposing memorisation. Otherwise *pseudonymous*.	Typically *minimal/limited* risk (research/education, not placed on market).	Not a medical device. No MDR.
U2. Pre-clinical model development/validation (bench/off-line)	Controller: AI developer/consortium. Processor: technical host and external labs.	Frequently *pseudonymous* unless strong evidence of anonymisation per Recital 26${}^{\text{e}}$.	If intended for healthcare diagnosis/triage: *high-risk* (pre-market stage of high-risk system).	If used to support clinical evidence for Software as Medical Device (SaMD): contributes to technical documentation and performance eval.
U3. Benchmarking/fairness audits across sites	Controller: benchmarking coordinator. Processors: participating sites’ IT.	Case-dependent; cross-site linkage may increase re-ID risk. Treat as *pseudonymous* unless tests show otherwise.	*Limited* to *high* depending on audited system’s intended medical purpose.	No MDR unless used as clinical evidence for a device.
U4. Training/validation for diagnostic AI to be CE-marked	Controller: manufacturer (defines purposes/means). Processors: CROs, cloud MLOps, annotators.	Treat as *pseudonymous* (linked to real distributions); document privacy tests; PETs recommended.	*High-risk* (health AI under MDR scope).	MDR applies: synthetic datasets must be traceable; included in clinical/performance evaluation package.
U5. Public release of synthetic dataset (open or controlled)	Controller: releasing institution (sets purpose/licence). Processor: repository/portal operator.	Release only if *anonymous* per Recital 26 with evidence: MIA/AIA≤baseline; NN/memorisation checks; small-population safeguards; documentation of pipeline.	Not an AI system per se; risk depends on downstream use.	No MDR by itself; downstream clinical use may trigger MDR.
U6. Cross-border secondary use via EHDS data access bodies	Controller: data user for secondary purpose; Data holder and access body have governance duties; Processors: data space infrastructure providers.	*Pseudonymous* by default; access body enforces safeguards. Anonymity must be justified with tests.	Depends on project: research (*minimal/limited*) vs. device development (*high-risk*).	If supporting intended clinical performance: MDR duties on the manufacturer (traceability, documentation).

${}^{\text{a}}$*EHDS role*: “controller” determines purposes/means; “Processor” acts on behalf of a controller. Data access bodies govern secondary-use access.

${}^{\text{b}}$*GDPR status*: “anonymous” only when re-identification is not reasonably likely considering time, cost, and technology; otherwise treat as “pseudonymous.”

${}^{\text{c}}$*AI Act risk*: healthcare diagnostic/therapeutic AI is generally high-risk. Research/education tools not placed on the market are typically minimal/limited risk.

${}^{\text{d}}$*MDR implications*: apply when software is a medical device or evidence contributes to a device’s clinical/performance evaluation.

${}^{\text{e}}$*Evidence expectations (Recital 26)*: membership/attribute inference attack results, nearest-neighbour/memorisation probes, small-population/rare-disease analysis, absence of linkage keys, and documentation of generation/DP/PET settings.

Table 2 translates typical synthetic-imaging use cases into concrete compliance expectations by aligning, for each scenario, (i) the likely EHDS roles (who is the controller defining purposes/means and who acts as processor), (ii) the GDPR posture (whether the dataset should be treated as anonymous, hence outside GDPR, or as pseudonymous, hence regulated), (iii) the anticipated AI Act risk categorisation given the intended use (e.g., research/education vs. diagnostic application), and (iv) any MDR implications where outputs support clinical performance or a medical device dossier. The entries are indicative and context-dependent, but they make explicit the evidence expected to justify an “anonymous” classification (e.g., attack metrics at or below baseline, absence of linkage keys, small-population safeguards) and when to default to a “pseudonymous” treatment with additional safeguards. Complementing the table, Figure 2 operationalises GDPR Recital 26 for synthetic imaging. It begins by asking whether the dataset was generated from personal data. If not, it is likely anonymous (outside GDPR) provided you document the generation process and confirm that no linkage keys exist. If it was generated from personal data, you then assess re-identification risk. First, run membership/attribute-inference attacks and compare their AUC to a random-guess baseline; results meaningfully above baseline indicate pseudonymous data (GDPR applies). Next, examine small-population/rare-disease signals (e.g., k-anonymity counts, site-specific rarity); any such risk also leads to a pseudonymous classification. Finally, check for linkage or memorisation risks—presence of keys, released weights/checkpoints that leak training samples, nearest-neighbour/inversion probes suggesting copying, or auxiliary datasets that enable linkage; any positive finding again implies pseudonymous treatment with safeguards. Only if all three checks (attacks, rarity, linkage/memorisation) are negative or at baseline may the dataset be deemed anonymous, in which case you retain the evidence demonstrating that re-identification is “not reasonably likely.” Dashed callouts in the figure list the concrete evidence expected at each step.

### Scholarly perspectives on synthetic data governance

A growing body of scholarship addresses the challenges and opportunities of synthetic data in the EU regulatory context. Scholars agree that while current frameworks allow for synthetic data use, legal ambiguity persists, especially regarding GDPR compliance (8). Synthetic data are increasingly recognised as pivotal for privacy-preserving data sharing under the EHDS, with recent advancements in deep generative models enhancing their fidelity and utility (14, 15). Nevertheless, concerns remain around re-identification risks. As Biasin et al. (29) and van der Wel (30) argue, synthetic data must be rigorously assessed based on how they are generated. Improperly designed datasets could still fall under GDPR provisions, particularly where re-identification is plausible. The GDPR’s flexibility allows Member States to implement additional rules for health data, resulting in regulatory fragmentation and potential obstacles to cross-border collaboration (23). Borissova (36) and Ciminá (37) highlight the tension between Open Science and data protection. While synthetic data offer a privacy-friendly alternative, their classification as anonymised or pseudonymised remains unclear, complicating their use in collaborative research. Cross-border data sharing further intensifies these challenges, particularly around consent, data sovereignty, and integrating privacy-preserving techniques such as differential privacy (15).

Debates also focus on the EHDS framework’s definition of health data. As noted by Foà (1), its scope includes both personal and non-personal data. Yet, it lacks clear guidance on how synthetic data should be classified. For Borissova (36), the legal uncertainty undermines the potential of synthetic data unless clarified within the EHDS.

International comparisons highlight regulatory divergence. Casarosa and Greser (32) contrasts the GDPR’s strict data protection with more permissive frameworks like HIPAA and China’s PIPL. These differences create barriers to interoperability and international collaboration, reinforcing calls by Borissova (36) for alignment through bilateral agreements or global governance initiatives.

In the technical domain, scholars advocate for using Privacy Enhancing Technologies (PETs) such as Federated Learning and Homomorphic Encryption to support secure data sharing (38, 39). These methods allow decentralised collaboration without raw data exchange, aligning with EHDS priorities and addressing data sovereignty concerns. The COVID-19 pandemic provided a testbed for such approaches, demonstrating the utility of synthetic data and the need for interoperable data models (14, 40).

Ethical and procedural transparency remains a central concern. Authors stress the importance of documenting synthetic data generation methods, publishing evaluation metrics, and developing meaningful consent frameworks—particularly when individuals are unaware that their data were used to generate synthetic datasets (20, 29, 36). Aspell et al. (41) argue that evaluation frameworks for synthetic data remain underdeveloped and call for robust metrics to assess fidelity, representativeness, and utility in applied healthcare contexts.

Regulatory misalignment further complicates implementation. The AI Act’s risk-based classification system does not neatly map onto GDPR principles, contributing to uncertainty around the legal status of synthetic data (31). Cybersecurity risks and unclear data ownership also pose challenges, with calls for international coordination to ensure ethical and lawful data use (32, 42). In particular, Davidson and Winter (43) underscore the importance of anticipating emerging governance gaps when multiple legal instruments—such as the GDPR, AI Act, and EHDS—intersect without clearly delineating responsibilities.

The lack of standardised interoperability protocols across Member States impedes the integration of synthetic data into cross-border health systems. Harmonisation of definitions and frameworks is critical to ensuring synthetic data fulfil their potential as safe, equitable tools for healthcare innovation (14, 36).

Synthetic data has an important role in the European digital health ecosystem. While current regulations do not explicitly address their use, synthetic datasets intersect with multiple legal frameworks, including the GDPR, AI Act, MDR, and EHDS. Scholarly contributions reveal a strong consensus on their potential to advance privacy-preserving innovation while highlighting unresolved issues surrounding legal classification, consent, interoperability, and ethical governance. Institutional efforts from the European Commission and scholarly calls for standardisation—such as those by Tozzi et al. (35), Pereira et al. (10), Davidson and Winter (43), and Aspell et al. (41)—underscore the importance of a harmonised and transparent framework. Addressing these challenges through coordinated regulation, technical standards, and ethical practices is essential to unlocking synthetic data’s full value in European healthcare and beyond.

## Synthetic image dataset methodology and case studies

Integrating synthetic data into medical imaging research within the European Health Data Space offers transformative potential but raises critical ethical concerns. This section discusses how synthetic data can address privacy preservation, data scarcity, and bias mitigation challenges. Three use cases are presented to illustrate cross-border collaboration in data transfer, the use of synthetic medical image datasets in validation, and the generation of data from Electronic Health Record (EHR) cases aligned with the objectives of the EHDS.

Ethical considerations include ensuring that synthetic datasets do not perpetuate or exacerbate biases present in the original data—particularly concerning under-represented populations—and maintaining representativeness across diverse demographic groups. Moreover, robust validation frameworks are essential to assess synthetic data’s reliability and clinical relevance, especially in sensitive domains such as healthcare diagnostics. Transparency in data generation processes and adherence to privacy regulations, such as the GDPR, foster trust among researchers, clinicians, and patients. By prioritising inclusivity, fairness, and accountability in synthetic data practices, the European Health Data Space can harness these innovative tools to advance medical imaging while upholding ethical standards essential for equitable healthcare innovation.

### Synthetic image data

Computer-Aided diagnosis (CAD), driven by AI and deep learning methods, has recently supported the process of medical image analysis—especially MRI—and diagnosis. However, training deep learning models requires large sets of medical imaging data. While Generative Adversarial Networks (GAN) were initially popular for generating synthetic data in the medical imaging domain, they continue to address this challenge effectively (5, 44). GAN are a class of deep learning models composed of two neural networks: a generator and a discriminator. The generator produces synthetic data by creating random noise and generating increasingly realistic outputs (44). At the same time, the discriminator evaluates whether the data are real or synthetic using binary classification (24, 44). The two networks improve iteratively through adversarial training, resulting in highly realistic synthetic outputs (45).

GAN have transformative applications in medical imaging datasets, especially for brain MRI. Clinically, they support advanced analysis by synthesising missing MRI sequences or generating 3D brain MRIs (45). GAN offer significant benefits across clinical research, medical education, and patient privacy protection, among other areas.

#### Synthetic data generation and diversity

The production of large volumes of synthetic medical images—such as realistic brain scans—can significantly enhance training datasets. This is especially valuable for rare conditions like brain tumours or neurodegenerative diseases, where real data is scarce (19, 44, 45). Synthetic data also benefits medical education by providing abundant training materials and interactive simulations, allowing students to learn more effectively and encounter a broader range of clinical scenarios. GAN, in particular, enable the creation of customised datasets that include both common and rare edge cases, helping students and professionals become familiar with diverse conditions (45). Moreover, synthetic medical image data is increasingly used to augment datasets for AI/ML-based diagnostic tools, predictive screening systems, and other health technologies. This helps mitigate challenges related to limited or imbalanced real-world datasets. Using synthetic data for pre-testing and validation can also reduce costs and accelerate research timelines before transitioning to real-world deployment (19). In summary, synthetic image data can support various aspects of medical research, education, and technology development. Synthetic image data can help with the following aspects:

1. Tumour segmentation
  Generative Adversarial Networks (GANs) have shown significant promise in enhancing tumour segmentation tasks by generating highly accurate segmentation masks that delineate tumour boundaries. This is particularly beneficial in brain MRI, where tumours often present with complex shapes and indistinct edges. By synthesizing annotated examples, GANs can augment training data and improve the robustness and sensitivity of segmentation algorithms used in clinical diagnostics (45).
2. Super-resolution and image quality enhancement
  Low-resolution or poor-quality medical scans can hinder diagnostic accuracy, particularly in resource-limited settings. GANs contribute to super-resolution by learning to enhance these images, producing higher-resolution outputs that retain anatomical fidelity. This refinement aids radiologists and AI systems alike in detecting subtle pathological changes that might otherwise be missed in noisy or compressed images (45).
3. Modality translation
  GANs can simulate disease progression by translating healthy brain MRIs into their diseased counterparts. This kind of image-to-image translation supports research and clinical training by creating synthetic but realistic progressions of neurodegenerative diseases, tumours, or stroke lesions. Such applications allow for controlled experimentation and help fill gaps where real-world longitudinal imaging data is limited or unavailable (45).
4. Prognosis and image registration
  Though less widely explored, GANs are emerging tools in prognosis modeling—predicting how a disease is likely to evolve over time based on imaging patterns. In addition, GANs assist in image registration by aligning images taken at different times or using different modalities, improving longitudinal analysis and multi-modal integration in treatment planning (45).
5. Protection of patient privacy
  Importantly, because synthetic images do not correspond to actual individuals, they eliminate the risk of patient re-identification. GAN preserve the statistical properties of real datasets without reproducing identifiable features, making them a powerful approach to data sharing that complies with ethical and legal standards such as GDPR and HIPAA (44).
6. Improved model performance
  Synthetic image data can substantially enhance the performance of machine learning models, especially in tasks where real data is limited or imbalanced. A notable study by Brugnara et al. (46) demonstrated this in the context of detecting new multiple sclerosis (MS) lesions on brain MRI. By integrating synthetic data into the training pipeline, the *ResNet* model’s AUC increased from 56% to 77.5%, while an attention-based model achieved a leap from 83.6% to 93.3%. These improvements underscore the potential of synthetic data in boosting generalisability and performance across different AI architectures and clinical settings.
7. Stress-testing AI models
  Synthetic data empowers researchers to replicate rare diseases and simulate challenging imaging conditions that are often underrepresented in real-world datasets. For instance, GANs can generate synthetic histological images of rare cancer subtypes or introduce artefacts such as noise and distortion, enabling robust testing of diagnostic models. Additionally, synthetic data plays a crucial role in mitigating domain shift. By emulating variations in imaging protocols, equipment types, and patient populations across different institutions, it helps uncover and correct biases in AI models trained on data from a single source. This significantly enhances the models’ ability to generalise across diverse clinical environments (24).

While a diverse array of methodologies exists for generating synthetic image data, our focus is on Generative Adversarial Networks (GAN) due to their initial popularity in the medical imaging domain (5, 44). Variations of GAN are particularly advantageous for preserving privacy while effectively generating synthetic tabular data. They are renowned for their ability to handle high-dimensional data; however, hyperparameter tuning is necessary to prevent model collapse (47).

The transformative role of GAN in medical imaging continues to expand, offering benefits that range from data augmentation to enhanced diagnostic precision and privacy preservation. Their versatility extends to generating synthetic data for rare conditions, improving image quality, simulating disease progression, and stress-testing AI models across varied imaging conditions. These capabilities not only enhance model robustness but also contribute to more equitable and generalisable clinical tools. To better understand the landscape of GAN applications in medical imaging, Table 3 summarises key GAN architectures and their corresponding use cases, drawing on recent findings by Kim et al. (5) and Cheng et al. (48).

**Table 3:** Different model of GAN for medical image synthetic data.

GAN model	Usage in medical imaging	Special features	Problem solved
Deep convolutional GAN (DCGAN)	Generates high-quality synthetic medical images, such as MRI or CT scans	Improves image quality and stability during training compared to earlier GAN	Addresses the need for high-quality synthetic data for training models, reducing privacy risks
Conditional GAN (CGAN)	Produces targeted medical images, such as those of specific diseases or orGAN	Enables controlled generation of images (e.g., specific tumour types)	Solves class imbalances in datasets by generating condition-specific data
Progressive growing GAN (PGGAN)	Creates high-resolution synthetic medical images, such as detailed brain MRIs	Stabilizes training and improves fine details in generated images	Addresses the challenge of generating high-resolution medical images needed for clinical applications
StyleGAN	Generates synthetic medical images with precise control over features like organ contours or tumor textures	Offers fine-grained control over high-level attributes (e.g., shape) and stochastic variations (e.g., texture)	Enhances diversity in datasets by allowing variation in image styles while maintaining realism
CycleGAN	Converts low-dose CT to high-dose CT or generates missing MRI sequences from other modalities	Works without paired datasets, using cycle-consistency loss to ensure realistic translations between modalities	Facilitates modality translation, reducing the need for multiple imaging sessions and radiation exposure
StarGAN	Synthesises multi-contrast MRI or echocardiography views from limited data	Handles diverse datasets and imaging modalities efficiently with a unified framework	Simplifies multi-modality imaging workflows and reduces dependency on large datasets across domains
Differentially private GAN	Generates privacy-preserving synthetic medical images but with reduced quality, making it less suitable for high-dimensional datasets like medical images	Protects against membership inference attacks, especially in scenarios with dataset shifts. Ensures privacy through DP-SGD, which adds noise and clips gradients during training	Addresses privacy concerns by preventing sensitive data leakage from training datasets. However, struggles with maintaining image quality and utility, which negatively impacts downstream classification tasks and fairness metrics

### Case studies in synthetic medical imaging

Several studies demonstrate the application of synthetic image datasets in overcoming barriers in medical research, particularly regarding privacy, data availability, and cross-border collaboration. Table 4 summarises selected case studies, their methodologies, and their impact on medical imaging.

**Table 4:** Summary of studies synthetising image datasets.

Reference	Used models	Problem addressed	Ethical considerations	Regulatory considerations	Key findings	Outcomes
Giuffrè and Shung (49)	GAN, VAE, differential privacy	Tackles privacy concerns by proposing privacy-preserving techniques like Differential Privacy (DP) to mitigate risks of re-identification in synthetic datasets. Also addresses data bias and quality issues for healthcare applications	Advocates for privacy-by-design approaches and risk mitigation strategies to ensure patient confidentiality	Points out limitations in GDPR and HIPAA for regulating synthetic data, emphasising the need for updated legal frameworks to address synthetic data risks comprehensively	Synthetic data can augment datasets for predictive analytics and improve data privacy while mitigating risks through DP and traceability measures like a chain of custody	Demonstrates that synthetic data can improve healthcare analytics but requires robust ethical frameworks and regulatory updates to ensure safe application
Eckardt (50)	CTAB-GAN+, NFlow	Addresses the lack of publicly available datasets for rare diseases like AML by generating synthetic cohorts that preserve inter-variable relationships and mimic real patient data distributions. Tackles privacy concerns with Hamming distance metrics	Ensures synthetic data does not allow re-identification of patients by quantifying privacy preservation using Hamming distances	Highlights the need for stringent regulatory oversight to enable the use of synthetic data in clinical trials while preventing misuse due to current gaps in GDPR and HIPAA regulations	Synthetic cohorts closely mimic real patient data in terms of demographics, molecular profiles, and survival outcomes, enabling their use in explorative analyses and as control groups in clinical trials	Provides proof-of-concept for using synthetic data in rare disease research, demonstrating high fidelity and usability while safeguarding patient privacy
Bottani et al. (25)	3D U-Net variants, conditional GAN	Resolves heterogeneity in clinical data warehouses by converting contrast-enhanced T1-weighted MRI (T1ce) into non-contrast-enhanced MRI (T1nce), enabling harmonised use of neuroimaging tools designed for T1nce images	Consent waived under French regulations for routine clinical care images	Adheres to local French regulations for using clinical data warehouses; emphasises the importance of ethical oversight when repurposing routine medical images for research purposes	Synthetic T1nce images are highly similar to real T1nce images and enable reliable feature extraction, demonstrating the potential to harmonize heterogeneous datasets from clinical warehouses	Demonstrates that deep learning models can harmonize heterogeneous imaging datasets, improving their usability in neuroimaging research while adhering to ethical and regulatory standards
Ntampakis et al. (51)	Conditional GAN, YOLOv8	Addresses the lack of robust evaluation frameworks for synthetic medical images by introducing a three-pronged evaluation strategy (expert domain assessment, statistical data analysis, adversarial evaluation). Focuses on synthetic pulmonary imaging for pneumonia diagnosis	Emphasises the importance of post-market evaluation to ensure clinical accuracy and reliability of synthetic images	Aligns with EHDS objectives by emphasising quality assurance and ethical use of synthetic data in medical imaging research	Introduces SPINE, a novel framework for evaluating synthetic pulmonary imaging. Demonstrates that synthetic data can achieve high fidelity to real-world data and be effectively used in machine learning for clinical diagnostics	Demonstrates that synthetic data can improve diagnostic accuracy and reliability in clinical applications when rigorously evaluated, setting a new standard for ethical and practical use in medical imaging
Angermann et al. (52)	3D-StyleGAN, GAN inversion techniques	Addresses limitations in understanding latent space properties of generative models for medical imaging applications by introducing latent space manipulation techniques like attribute editing and style mixing	Ethical considerations focus on ensuring that generated images maintain clinical relevance and do not introduce bias or artifacts that could mislead diagnostic algorithms	Emphasis on improving dataset diversity and augmenting small datasets aligns with EHDS objectives to foster innovation through shared health data resources across Europe	Demonstrates that latent space manipulation can be used to customize synthetic medical images for specific attributes (e.g., trabecular or cortical bone density), enhancing their utility in research and clinical applications	Opens new possibilities for customizing synthetic datasets to address specific research needs or augment small datasets, supporting innovation in medical imaging research while adhering to ethical guidelines
Lara et al. (26)	Conditional GAN (CGAN), ResNet, attention-based CNN	Tackles the issue of domain shift in AI models caused by variability in MRI acquisition protocols, scanner types, and field strengths. Uses synthetic FLAIR images generated via CGAN to augment datasets and improve AI model performance on unseen data	Ethical approval obtained from Heidelberg and Bonn University Hospitals; informed consent waived due to retrospective nature of the study. emphasises the importance of rigorous external validation to ensure fairness and reliability	Aligns with EHDS objectives by addressing interoperability challenges and improving AI generalisability across institutions, which supports cross-border collaboration in medical imaging research	Synthetic data augmentation significantly improved AI model performance on external MRI datasets (AUC: 83.6% without synthetic data vs. 93.3% with synthetic data; p = 0.03). Demonstrates that synthetic data can mitigate domain shift and enhance model robustness	Demonstrates that synthetic data can improve AI model generalizability across heterogeneous datasets, making it a valuable tool for clinical applications where real-world data variability is a challenge
Vaden et al. (53)	Multiple imputation, statistical modelling	Addresses the challenge of sharing neuroimaging data while preserving patient privacy. Proposes a fully synthetic data generation approach to replicate group-level findings without including any real patient data, reducing re-identification risks	Ethical approval obtained; emphasises the importance of privacy-preserving synthetic datasets to mitigate risks of re-identification. Discusses the need for transparency in sharing synthetic data with appropriate safeguards	Aligns with EHDS objectives by enabling secure data sharing across institutions while maintaining compliance with privacy regulations like GDPR	Fully synthetic neuroimaging datasets accurately replicate group-level statistical results (e.g., variance, covariance, and voxel-level associations) while significantly reducing privacy risks compared to de-identified real-world datasets	Demonstrates that fully synthetic neuroimaging datasets can facilitate scientific replication, exploration, and education while minimizing privacy risks and enabling broader data-sharing initiatives in compliance with ethical and regulatory standards
Arora and Arora (54)	Progressively growing GAN (PGAN), Convolutional Neural Networks (CNNs)	Addresses the challenges of limited and diverse datasets in retinopathy of prematurity (ROP) diagnosis due to privacy concerns, disease rarity, and label inconsistencies by generating synthetic retinal vessel maps (RVMs)	Ethical approval obtained from multiple institutional review boards; written informed consent was obtained from parents of enrolled infants. Ensures that synthetic data does not reveal Protected Health Information (PHI)	Aligns with EHDS goals by enabling privacy-preserving data sharing and collaboration across institutions, supporting cross-border research and innovation	Synthetic RVMs generated by PGAN improved CNN performance for plus disease detection, achieving a higher AUC (0.971) compared to CNNs trained on real RVMs (AUC = 0.934). Synthetic data spanned the disease severity spectrum and preserved privacy by being distinct from training data	Demonstrates that synthetic datasets can enhance model performance, improve dataset diversity, and preserve patient privacy, making them a viable alternative for training robust AI models in medical imaging while addressing ethical and privacy concerns
Arora and Arora (54)	Bayesian regression, random forest, neural networks	Examines whether synthetic data can replace real data for training machine learning models to predict blood pressure, addressing privacy concerns and limited access to sensitive health data	Ethical approval obtained; emphasises the importance of privacy-preserving synthetic datasets to mitigate risks of re-identification	No explicit mention of EHDS but aligns with its goals by enabling privacy-preserving data sharing and fostering cross-border research and innovation in healthcare	Models trained on synthetic data performed comparably to those trained on real data, with no significant differences in accuracy (MAE: 8.12–8.33). Synthetic datasets demonstrated high fidelity with real data	Demonstrates that synthetic data can be a viable substitute for real data in training machine learning models, preserving privacy while maintaining performance
Pereira et al. (55)	Marginal-based synthesisers (AIM, MWEM PGM), GAN-Based Synthesisers (DP-GAN, PATE-GAN)	Investigates the utility and fairness of differentially private synthetic data in machine learning pipelines for tabular datasets, focusing on classification tasks and subgroup fairness metrics	Highlights the importance of balancing utility and fairness in synthetic datasets while ensuring compliance with privacy laws through differential privacy guarantees	Supports EHDS objectives by enabling secure sharing of private health data and ensuring fairness in AI applications across diverse populations	Marginal-based synthesizers (e.g., AIM, MWEM PGM) outperformed GAN-based synthesizers in both utility and fairness metrics. AIM synthetic data achieved AUC scores comparable to real data across multiple datasets, preserving fairness characteristics	Demonstrates that marginal-based synthetic datasets can train machine learning models with utility and fairness comparable to real data, making them suitable for privacy-preserving applications in healthcare research
Pozzi et al. (56)	Diffusion models (U-Net architecture)	Addresses challenges in generating high-quality synthetic pathology images for digital pathology tasks, including tissue classification and addressing data scarcity while preserving patient privacy	Ethical considerations include ensuring biological realism of generated images and mitigating risks of creating clinically irrelevant artifacts through expert validation by pathologists	Aligns with EHDS goals by facilitating secure sharing of anonymised medical imaging datasets across institutions while maintaining clinical relevance	Diffusion models outperformed GAN in generating high-quality synthetic pathology images across five tissue types. The generated images demonstrated high similarity to real images (measured by FID) and were validated as biologically realistic by expert pathologists	Provides a comprehensive pipeline for generating and evaluating synthetic pathology images, demonstrating their usability for deep learning tasks while ensuring clinical relevance through multifaceted evaluation methods
Yan et al. (57)	GAN (EMR-WGAN)	Addresses limited accessibility of electronic health records (EHRs) due to privacy concerns, enabling broader use of health data for machine learning and biomedical applications	Highlights the importance of privacy risk mitigation and fairness across patient subpopulations in synthetic EHR data	Discusses GDPR and other frameworks but emphasises the need for tailored regulatory measures for synthetic health data	Synthetic EHR data can enhance machine learning model performance, reduce biases, and support hypothesis generation while maintaining privacy	Provides a tutorial demonstrating how GAN-based models can generate high-quality synthetic EHRs for research, education, and AI development
Vaden et al. (53)	Multiple imputation model, 1. Synthetic predictor tables (MICE R-package); 2. Synthetic Gray matter images	Solves challenges in sharing neuroimaging data by creating fully synthetic datasets that replicate group-level findings while minimising re-identification risks	Focuses on limiting re-identification risks, particularly for sensitive populations like paediatric cases, through fully synthetic datasets	Addresses shifting regulatory landscapes but does not explicitly reference specific regulations like GDPR	Fully synthetic neuroimaging datasets accurately replicate statistical properties of observed data while reducing privacy risks	Demonstrates that fully synthetic neuroimaging datasets can facilitate replication, exploration, and education without compromising individual privacy
Aunón et al. (39)	Differential privacy, federated learning, homomorphic encryption	Addresses challenges in data sharing across organizations while ensuring privacy and compliance with regulations. Focuses on enabling secure collaboration in data spaces	Emphasises privacy preservation through technologies like encryption and federated learning to mitigate risks of re-identification and misuse of shared data. Advocates for ethical use of shared data, respecting individual rights	Refers to GDPR for data protection, highlighting the role in shaping privacy-enhancing technologies for data spaces	Privacy-enhancing technologies can enable secure collaboration in data spaces while maintaining compliance with regulations and protecting sensitive information	Demonstrates that adopting privacy-enhancing technologies facilitates secure and compliant data sharing across organizations, fostering innovation while safeguarding privacy
Schütte et al. (58)	Progressive GAN (Prog-GAN), conditional projection-based GAN (CPD-GAN)	Addresses privacy concerns in medical data sharing by generating synthetic datasets that retain statistical properties of real data without revealing sensitive information	Highlights the importance of privacy-preserving synthetic data to prevent patient re-identification	Suggests synthetic datasets as an alternative to comply with GDPR and other privacy regulations on medical data sharing	Synthetic data can match real data performance for predictive tasks under specific conditions but faces challenges like label overfitting and reduced quality at high resolutions	Synthetic medical images can be a viable alternative for privacy-preserving data sharing, but improvements are needed for high-resolution image generation
Coyner et al. (59)	Progressively growing GAN (PGAN)	Tackles challenges in training AI models for rare diseases like retinopathy of prematurity due to limited and diverse datasets and privacy concerns	Emphasises the reduced risk of re-identification with synthetic images, allowing broader data sharing	Proposes synthetic datasets as a privacy-preserving method that avoids reliance on protected health information	CNNs trained on synthetic retinal vessel maps outperformed those trained on real data in detecting plus disease, demonstrating the utility of synthetic datasets	Synthetic datasets enhance model robustness and diversity while maintaining diagnostic accuracy, offering a solution for rare disease research and privacy-preserving collaborations
Zheng et al. (60)	PGGAN, DL models	Addresses the lack of diverse and well-labelled datasets for training deep learning models in detecting angle closure in anterior segment optical coherence tomography (AS-OCT) images	Ensures privacy by generating synthetic AS-OCT images to avoid sharing real patient data, reducing risks of re-identification	Highlights the need for ethical and legal frameworks to regulate synthetic data usage in medical imaging applications	Synthetic AS-OCT images generated by GAN are of high quality and can train deep learning models with diagnostic performance comparable to real images, achieving AUCs of 0.97 and 0.94 in validation datasets	Demonstrates the potential of synthetic data to augment deep learning model training and improve diagnostic accuracy for ophthalmological conditions such as angle closure
Krause et al. (61)	CGAN, shuffleNet	Addresses the shortage of publicly available histology images with matched molecular data for training deep learning models to detect microsatellite instability (MSI) in colorectal cancer (CRC)	Synthetic histology images reduce ethical concerns by avoiding patient-specific data sharing, while retaining latent genetic information about molecular alterations	Emphasises the importance of ethical guidelines and regulatory frameworks for synthetic data sharing and usage in digital pathology research	Synthetic histology images retain genetic information and can train deep learning models for MSI detection with performance comparable to real image-based systems, achieving AUROC values up to 0.777 when combining real and synthetic datasets	Demonstrates that synthetic histology images can augment or replace real datasets, enabling scalable AI systems for genetic profiling while addressing privacy concerns effectively

### Case studies

Two use cases from Aunón et al. (39) and Yan et al. (57) have been selected as case studies due to their strong alignment with the objectives of the EHDS, particularly in enabling secure cross-border data sharing and reuse, while addressing challenges related to privacy and interoperability.

Aunón (39) comprehensively evaluate Privacy-Enhancing Technologies (PETs), including Federated Learning, Differential Privacy, and Homomorphic Encryption. It demonstrates their applicability within data spaces to support secure collaboration across organisations. The study emphasises the importance of privacy preservation in facilitating data sharing, directly resonating with the EHDS’s goal of establishing interoperable and secure frameworks for health data exchange across the EU and beyond. Furthermore, it highlights practical examples—such as the application of federated learning in healthcare use cases—showing how PETs can help overcome critical barriers like data heterogeneity, quality issues, and privacy concerns, which are key challenges to fulfilling the EHDS’s vision.

In contrast, Yan et al. (57) focus on generating synthetic Electronic Health Record (EHR) data using advanced GAN-based models, such as EMR-WGAN. This paper addresses the EHDS’s objective of standardising and making health records interoperable by offering a transparent tutorial on generating high-quality synthetic EHRs that preserve statistical fidelity while protecting patient privacy. The practical application of synthetic data generation supports secondary uses, including machine learning model development, hypothesis generation, medical education, and AI-driven healthcare innovation. Unlike other studies that concentrate solely on privacy or technical implementation, this paper illustrates how synthetic EHR data can be tailored to specific use cases while remaining accessible and usable across various healthcare systems.

These two papers stand out by offering actionable frameworks that align with EHDS regulatory aims while providing practical solutions to technical barriers in health data sharing. Aunón (39) and Yan et al. (57) uniquely address both technological maturity—such as federated learning protocols—and real-world implementation—such as GAN-based synthetic EHR generation—making them exemplary for illustrating how EHDS objectives can be effectively operationalised across Europe and on a global scale.

#### Case study 1: cross-border synthetic data transfer

The primary objective of this experiment was to demonstrate how Federated Learning (FL) facilitates collaborative machine learning across multiple healthcare institutions while preserving patient privacy. It aligns with the broader aims of health data spaces, which seek to enable secure and privacy-preserving data sharing to advance medical research and enhance patient outcomes. By leveraging FL, the study tackles critical issues such as data accessibility, heterogeneity, and privacy concerns, showcasing FL’s potential to overcome major barriers in the healthcare domain.

The study employed publicly available datasets of skin lesion images sourced from four institutions in Australia, Austria, Brazil, and the Netherlands. The datasets included HAM10000, PAD-UFES-20, and MED-NODE. Initially, the combined dataset contained eight types of skin lesions, but this was reduced to five (nevus, melanoma, actinic keratosis, basal cell carcinoma, and benign keratosis) due to class imbalance and insufficient sample sizes for certain lesion types. This selection helped mitigate data imbalance by focusing on lesion categories that were adequately represented across the participating institutions.

Several preprocessing steps were carried out to prepare the data for training. First, the HAM10000 dataset was split into subsets corresponding to Australia and Austria, creating four distinct datasets. The images were then normalised to a resolution of 224 × 224 pixels with RGB channels. Each pixel value was standardised by subtracting the mean and dividing by the standard deviation of each channel. Finally, each dataset was divided into training (75%) and testing (25%) sets, ensuring no overlap. A global test set was constructed from the local test sets to assess the generalisation performance of the FL model.

A pre-trained MobileNet v2 Convolutional Neural Network (CNN) was utilised for transfer learning due to its proven effectiveness in skin lesion classification tasks. Transfer learning reduced the need for extensive labelled data and minimised computational requirements. A weighted cross-entropy loss function was applied to address class imbalances in this multiclass classification task. Class weights were set inversely proportional to the sample sizes of each class, ensuring that under-represented classes had more significant influence during model training. For the FL setup, local models were trained independently on each dataset for 50 epochs with a batch size of 32, using stochastic gradient descent (SGD) and a learning rate 0.001. Models were then aggregated using the FedAvg strategy over 20 communication rounds, each involving 10 epochs of local training per institution.

The performance of both local and federated models was assessed using two key metrics: the F1 score and confusion matrix analysis. F1 scores were calculated per class and as a weighted average across all classes to account for class imbalance. The confusion matrix was normalised by class support to provide a clear visualisation of prediction accuracy for each class.

The findings revealed significant differences between models trained locally and those trained using the federated approach. Locally trained models performed well on lesion types sufficiently represented in their respective datasets but showed poor performance on under-represented classes or those absent from the local data. For instance, the Netherlands dataset lacked certain lesion types, resulting in zero predictive capability for those classes.

In contrast, the federated model demonstrated improved generalisation across all lesion types. Notably, institutions such as the one in the Netherlands could predict lesion types absent from their local training data due to knowledge transfer through model aggregation. However, slight reductions in performance were observed in specific cases—for example, predictions of actinic keratosis in Australia—likely due to averaging effects inherent in the aggregation process. By sharing model parameters instead of raw data, FL preserved patient privacy while enabling collaborative learning across diverse and geographically distributed datasets.

This experiment highlights the transformative potential of FL for healthcare data sharing within health data spaces. It shows that FL can address challenges such as data heterogeneity, class imbalance, and privacy in multiclass classification problems. It also underscores the importance of preprocessing and strategically handling imbalanced datasets for successful model training. From an industry perspective, FL facilitates collaborative research without compromising data ownership or patient confidentiality. It enables wider access to diverse datasets, thereby improving the robustness and generalisability of AI models. Future directions include integrating FL with other PET—such as Differential Privacy or Secure Multi-Party Computation—to bolster security and reduce risks like potential information leakage from model updates. Furthermore, developing advanced aggregation techniques tailored to heterogeneous datasets could further enhance performance in federated environments.

In conclusion, this case study demonstrates how FL aligns with the core objectives of health data spaces by enabling secure, privacy-preserving collaboration among healthcare institutions while addressing essential challenges in AI-driven medical research.

#### Case study 2: synthetic electronic health record

The primary objective of this experiment (included as non-imaging comparator to clarify governance issues that equally affect imaging) was to create high-quality synthetic EHR data that mimics the statistical properties of real-world patient data while maintaining privacy. It aligns with the objectives of the EHDS, which seeks to promote secure and privacy-preserving data sharing to advance healthcare research and innovation. This approach addresses key challenges in health data accessibility by enabling the generation of realistic synthetic datasets, including privacy concerns, data scarcity, and imbalances in the subpopulation representation.

**Figure 3:**
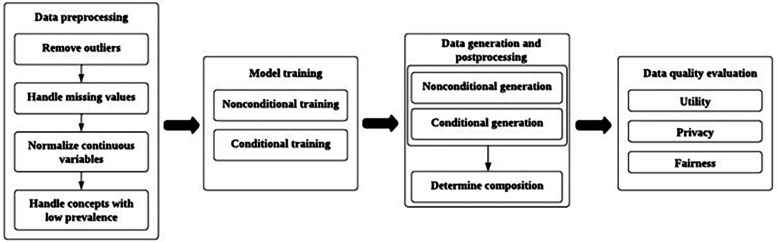
An overview of synthetic electronic health record data generation process through training generative models (Yan et al., 2024).

This study utilised the publicly available MIMIC-IV dataset from the Beth Israel Deaconess Medical Centre, which contains structured EHR data from patients admitted to intensive care units between 2008 and 2019 (Figure 3). The dataset includes demographic information, diagnoses, procedures, and clinical measurements such as BMI and blood pressure. A subset of patients with at least one hospital admission was extracted, resulting in a cohort of approximately 181,000 patients. The diagnoses were converted from ICD-9/10 codes to phecodes for clinical relevance and dimensionality reduction.

Preprocessing involves several steps to prepare the data for GAN training. Outliers were identified and removed based on clinically implausible values (e.g. BMIs > 60 or < 10). Missing values were addressed using random sampling based on marginal distributions for variables, such as BMI and blood pressure, which had around 38%–43% missing rates. Continuous variables were normalised to a range of (0,1) to ensure consistent scaling during model training. Low-prevalence concepts were removed or aggregated into higher-level categories to improve the model performance while maintaining clinical relevance.

The GAN architecture consists of two neural networks: a generator that produces synthetic records and a discriminator that distinguishes between real and synthetic data. The EMR-WGAN model was employed because of its ability to capture complex statistical relationships in EHR data while stabilising the training through Wasserstein divergence. The training used a non-conditional paradigm where all variables were treated equally without explicit labels guiding the generation. Multiple runs were performed with checkpoints evaluated for optimal synthetic data quality to address challenges, such as mode collapse and instability in GAN training. A SoftMax layer was added to ensure that categorical variables adhered to the one-hot encoding constraints. While record-level clinical constraints (e.g. prohibiting male patients from having female-specific diagnoses) were not enforced during training, these violations were analysed during evaluation.

The quality of the synthetic data was assessed across three dimensions: utility, privacy, and fairness. The utility metrics include:

• Dimension-wise Distribution: Evaluated how well synthetic data preserved the variable distributions.
• Column-wise Correlation: Measured correlation consistency between real and synthetic variables.
• Latent Cluster Analysis: Assessed structural similarity in the latent space.
• Prediction performance: Comparison of model performance when trained on real vs. synthetic data.
• Feature Importance: Examined overlap in key predictive features.

Privacy risks were evaluated using membership and attribute inference attacks, whereas fairness focused on equitable representation across patient subpopulations. The results demonstrated that the EMR-WGAN model effectively generated high-quality synthetic EHR data while reducing privacy risks compared with real datasets. Dimension-wise distribution analysis showed that the second run achieved the lowest absolute prevalence difference (APD), indicating better preservation of variable distributions. Column-wise correlation and latent cluster analysis also highlighted strong alignment with real data structures.

However, this study had some limitations. Male-specific diagnoses were occasionally assigned to female records in synthetic datasets because of the insufficient preservation of sex-diagnosis correlations. Privacy evaluations revealed significantly reduced membership inference risks compared to real data but highlighted minor variations across runs. This experiment underscores the potential of GAN-based synthetic EHR generation as a transformative tool for healthcare research within the EHDS framework. This approach addresses critical barriers, such as privacy concerns and limited access to diverse patient populations, by enabling the secure sharing of realistic yet anonymised datasets. Synthetic EHRs offer opportunities for hypothesis testing, model training, and educational purposes without compromising patient confidentiality. In the health care industry, they facilitate software development, medical education, and system testing under realistic conditions.

Future directions include incorporating temporal information into synthetic records for longitudinal analyses, improving fairness across subpopulations, and exploring hybrid models that combine GAN with other privacy-enhancing technologies such as differential privacy. These advancements align with the EHDS goals by promoting equitable access to high-quality health data while safeguarding individual privacy.

### Failure modes and a minimum validation battery for synthetic medical imaging

From methodological overview to quality assurance, we now specify imaging-specific failure modes and a minimum validation battery. Although synthetic data can mitigate access and privacy barriers, generative pipelines are prone to well-documented pitfalls, such as mode collapse and diversity loss, identity leakage/memorisation that elevates re-identification risk, overfitting and site/style shortcuts that undermine external validity, and hallucinated anatomical artefacts that erode clinical credibility. Because these issues directly intersect with the privacy (e.g., membership/attribute inference) and fairness concerns discussed earlier, a clear set of pre-release checks is needed before any secondary use under the EHDS. Below, we enumerate imaging-specific failure modes and propose a minimum validation battery that covers utility/fidelity on held-out real data, privacy attack testing, subgroup fairness, and documentation/traceability, establishing a pragmatic evidentiary floor. This battery is designed to complement the regulatory mapping (Table 2, Figure 2) and to operationalise the governance dimensions formalised in the SID-GT toolkit, enabling data stewards, developers, and access bodies to apply consistent acceptance criteria aligned with EHDS objectives.

#### Minimum validation battery before secondary use under EHDS

We recommend that the following *baseline battery* be completed and reported before secondary use or sharing within EHDS. Items map to the governance pillars (utility, privacy, fairness, traceability, and disclosure) and to EU frameworks, as summarised elsewhere in the manuscript.

**A. Utility & fidelity (held-out real data)**

• *Task performance on held-out real data*: report AUC/F1, sensitivity/specificity, and calibration (e.g., ECE/Brier), comparing (i) real-only, (ii) real+synthetic, and (iii) synthetic-only training.
• *External/site validation*: evaluate across at least one external site/scanner/protocol to test for domain shift; include ablations of the synthetic proportion.
• *Expert review*: double-blinded radiologist rating (e.g., 5-point realism/anatomical plausibility) with inter-rater agreement (e.g., Cohen’s κ); flag systematic artefacts.
• *Distributional alignment*: report simple but informative shifts (intensity histograms, lesion size/location distributions); include nearest-neighbour distance distributions to detect collapse.

**B. Privacy (link to “Privacy Risks and Re-identification”)**

• *Membership/attribute inference*: report attack AUC vs. random baseline; describe attacker knowledge. If above-baseline, treat as *pseudonymous* and apply additional safeguards.
• *Memorisation probes*: nearest-neighbour search in a perceptual feature space; duplicate detection; generator inversion tests; report minimum distances and exemplar pairs.
• *If using differential privacy (DP)*: report ϵ,δ, clipping/noise schedule, and induced utility trade-offs; document residual risks in the release notes.

**C. Bias & fairness (link to “Bias and Fairness”)**

• *Subgroup metrics with uncertainty*: report AUC/F1 and calibration with 95% CIs for sex, age bands, ethnicity (where lawful), and site; pre-specify acceptable disparity margins (e.g., ΔAUC within a narrow, justified range).
• *Coverage summary*: provide subgroup counts/percentages in the synthetic set; describe any targeted augmentation or reweighting applied.

**D. Traceability**

• *Data/model cards*: document generator architecture, training data provenance and inclusion/exclusion criteria, PETs/DP settings, seeds/checkpoints, and versioning; maintain chain-of-custody records.

**E. Disclosure & labelling**

• *Synthetic labelling & intended use*: mark datasets as synthetic; state intended scope (research, education, pre-clinical validation), limits (e.g., under-represented phenotypes), and residual risks. Provide the licence and contact.

#### Acceptance guidance

• *Release for broader secondary use (e.g., public/consortium sharing)*: only if attack metrics are at or near baseline, no memorisation evidence is found, expert review shows no systematic anatomical artefacts, and subgroup disparities are within pre-specified margins. Otherwise, treat as regulated pseudonymous data with restricted access and safeguards.
• *Clinical evidence contribution*: when synthetic data contributes to device performance documentation, ensure full traceability and external validation; prominently disclose synthetic proportions and any fairness mitigations.

This validation battery is designed to be used in conjunction with the governance checklist (SID-GT, cf. Table 5), providing a pragmatic baseline for quality, privacy, and equity before secondary use under EHDS.

**Table 5:** SID-GT: Pillars, reporting checklist, and EU framework mapping.

Pillar	What to report (checklist)	EU framework anchors
1. Utility & fidelity	Held-out *real-data* performance (AUC, F1, sensitivity/specificity, calibration); task definition and clinical/contextual thresholds; external validation/site-shift tests; ablation of synthetic-vs-real mix; data leakage checks.	*AI Act* (Art. 10–15 data governance, risk mgmt., transparency); *MDR* (clinical evidence/performance evaluation for intended purpose).
2. Privacy	membership- and attribute-inference attack AUC vs. random baseline; nearest-neighbour/memorisation probes; if DP used: report ϵ,δ, clipping/noise schedule, and utility trade-off; residual linkage risks (singling out/linkability/attribute inference) and mitigations.	*GDPR* (Recital 26; Arts. 4, 5, 25 privacy by design); *EHDS* (secondary-use safeguards, access governance).
3. Bias & fairness	Subgroup metrics (sex, age, ethnicity, site) with CIs; ΔAUC/ΔF1 vs. overall; calibration by subgroup; dataset composition/coverage; mitigations (reweighting, fairness-aware synthesis, targeted augmentation) and post-hoc audits.	*AI Act* (Art. 10 data quality/representativeness); *EHDS* (equitable access/quality).
4. Traceability	Model/data cards: purpose, provenance, training recipe, PETs, hyperparameters; versioning and chain of custody; seeds/checkpoints; deterministic build info; interoperability notes (schemas/ontologies).	*AI Act* (Art. 12 record-keeping/technical documentation); *MDR* (Annex II technical file); *EHDS* (interoperability/metadata).
5. Disclosure & labelling	Explicit “synthetic” labelling; intended use (research, education, pre-clinical validation); limits and residual risks; licence/usage restrictions; contact for queries; dataset DOI/version.	*GDPR* (Arts. 12–14 transparency); *EHDS* (access & transparency obligations).

## Critical discussion and future directions

### Current challenges

The rapid advancement of AI in healthcare presents significant ethical, technical, and regulatory challenges. As Federico and Trotsyuk (62) argue, there is a dual obligation to foster innovation while safeguarding individual rights, with particular attention paid to unintended consequences, such as privacy breaches and the amplification of existing biases. Their call for a globally harmonised regulatory framework reflects a growing consensus that balancing innovation with public welfare requires coordinated, anticipatory governance. A similar perspective is echoed by Aucouturier and Grinbaum (63), who advocates a shift from compliance-driven assessments to ethics-by-design methodologies. Such an approach would embed ethical considerations directly into the development pipeline of AI systems, thereby enhancing their long-term accountability and societal alignment. Collaborative governance is critical in this context. Baumgartner et al. (15) emphasise the importance of interdisciplinary teams in managing the complexities of modern health data systems, particularly where data sovereignty, interoperability, and privacy intersect. Colonna and Submitter (64) further note that as private actors increasingly influence public AI research, governance frameworks must evolve to counter regulatory arbitrage and ensure that societal interests are prioritised over commercial gain.

Cross-national data sharing introduces additional complexity owing to the heterogeneous nature of datasets. When data originate from multiple countries, format, quality, and completeness variations can negatively impact the performance of privacy-enhancing technologies (PETs). This highlights the need for rigorous pre-processing and homogenisation techniques. He (2) points out that without a clear EU-level interpretation of relevant laws, data access bodies and holders may apply inconsistent standards, potentially undermining the EHDS goals. Therefore, EHDS implementation must be closely monitored to ensure that data minimisation and ethical use are consistently upheld. The quality and representativeness of datasets remain a persistent concern. Biasin et al. (29) and Burden et al. (38) warn of the dangers of “data contamination” from synthetic content and stress the need for diverse, high-quality datasets to prevent AI systems from reinforcing existing inequities. Without rigorous standards and continuous auditing, AI-driven systems risk perpetuating harmful biases and eroding public trust.

Transparency is a fundamental element in building trust. Baumgartner et al. (15) underscore the value of open-source methodologies and documentation for fostering confidence in synthetic data and AI applications. However, as Federico and Trotsyuk (62) cautioned, existing regulations often fail to enforce transparency, leaving significant gaps in accountability. Another pressing challenge relates to the resource demands of synthetic data generation, particularly when using models, such as GAN, for medical image synthesis. As Arora and Arora (44) noted, the generation of high-quality synthetic images requires substantial computational power. Asadi et al. (65) similarly highlights the significant trade-offs between the costs of training GAN measured in time, energy, and memory, and the practical benefits of using synthetic data. This raises a critical question: are the performance gains from synthetic datasets sufficient to justify their resource intensity?

### Future directions for synthetic data

The future of AI in healthcare will depend mainly on addressing the aforementioned challenges, particularly regarding the ethical, technical, and governance aspects of synthetic data. Bertl et al. (66) identified several key barriers, including a lack of standardised interoperability frameworks, ethical uncertainties in data reuse, and insufficient collaboration among stakeholders. These challenges are exacerbated by the speed of technological change, which often outpaces the adaptability of the existing regulatory frameworks. Therefore, robust yet flexible standards are needed to ensure AI systems remain equitable, safe, and effective (15, 62, 64, 66).

An equally pressing concern is the fair distribution of the benefits of AI. Federico and Trotsyuk (62) stressed the importance of ensuring that AI advancements serve both underrepresented and underserved populations. Their view aligns with that of Aucouturier and Grinbaum (63), emphasising ethical governance structures that prioritise inclusivity and societal well-being. The challenges and opportunities surrounding synthetic medical imaging within the EHDS framework require a coordinated set of actions. These must address technical hurdles and the ethical and legal complexities of the implementation. Central to this effort is the need to clarify data ownership, especially when synthetic datasets are derived from personal health data. Intellectual property concerns must be resolved, and individuals should retain agency over how their real or synthetic data are used. It includes the development of harmonised consent frameworks that enable patients to make informed decisions about their data and provide mechanisms to withdraw consent when needed.

Bias mitigation must also be prioritised in synthetic data development. This can be achieved by integrating fair auditing and demographic balancing mechanisms into the data generation pipeline. Ensuring that synthetic datasets reflect a wide range of populations is critical for achieving equitable AI outcomes. Interoperability is another major hurdle to overcome. Unified protocols and standards across EU member states are essential for facilitating seamless data exchange while respecting national data sovereignty. Harmonising these frameworks will be key to enabling collaborative AI innovation throughout Europe. To prevent fragmentation and inconsistency, the EU must take the lead in establishing a unified standard for synthetic-data governance. It could be modelled after initiatives such as FUTURE-AI, which propose benchmarks for technical quality, ethical integrity, and interoperability. Such frameworks should promote transparency, fairness, and robustness, which are essential criteria for ensuring the reliability of synthetic data and their integration into clinical workflow.

Embedding ethics-by-design principles at every stage of synthetic data development is vital. From the outset, privacy, consent, and fairness should be considered and supported by the interdisciplinary ethics committees. Regular ethical audits should be institutionalised to address risks and proactively build public trust. Transparency in the data-generation process is crucial. Developers should provide detailed documentation of their methodologies, algorithms, and validation metrics. Open sourcing of these frameworks, where possible, would facilitate peer review, replication, and broader collaboration, aligning with Open Science principles and enhancing trust in synthetic data tools. In essence, ethical frameworks should not be viewed as regulatory hurdles but as enablers of trust and innovation. Within EHDS, robust governance mechanisms are essential for unlocking the full potential of synthetic imaging data. Addressing consent, ownership, bias, and interoperability issues is fundamental to this vision. Synthetic data offers the potential to transform healthcare by enabling secure, privacy-preserving data sharing and supporting the development of AI applications that are accessible and equitable. With careful alignment of technical innovation and ethical regulations, synthetic data can support advances in diagnostics, personalised treatment, and public health, while maintaining public confidence and compliance with fundamental rights.

One of the most exciting developments in this space is the integration of synthetic medical image generation with natural language processing (NLP), mainly through vision-language models (VLM). Systems such as LLaVA-Med and Med-PaLM merge the interpretive strengths of computer vision and text-based AI to unlock powerful new applications (5):

• Guided image synthesis: VLM can create synthetic medical images based on clinical text inputs. For example, given a description such as “an MRI scan showing glioblastoma with a 5 cm lesion in the frontal lobe,” the system can generate a corresponding synthetic image.
• Multimodal dataset creation: By pairing synthetic images with automated text annotations, VLM can create large-scale multimodal datasets for tasks such as classification, segmentation, or anomaly detection.
• Visual question answering (VQA): These models can generate synthetic visual responses to clinical queries, thereby supporting medical education and training by providing tailored examples.
• Cross-modality synthesis: VLM facilitate conversion across imaging modalities (e.g. MRI to CT), supporting diagnostic flexibility, and reducing redundant scanning.
• Automated annotation and captioning: VLM streamline the process of labelling images by generating consistent, detailed captions, saving time and ensuring annotation quality.
• Interactive data generation: Through conversational interfaces, clinicians can iteratively refine synthetic images by adjusting input specifications, enhancing usability, and tailoring outputs to clinical needs (5).

### Towards a governance toolkit for synthetic imaging data

While the ethical and regulatory considerations discussed above provide a conceptual foundation, their translation into actionable practices remains a key challenge. To support alignment with the European Health Data Space (EHDS) objectives, we propose a Synthetic Imaging Data Governance Toolkit (SID-GT). This toolkit serves as a practical checklist for researchers, developers, and data custodians to evaluate synthetic datasets along five essential governance pillars: utility, privacy, fairness, traceability, and disclosure. The toolkit and checklist presented here target synthetic medical imaging; when guidance generalises beyond imaging, this is indicated explicitly. Each pillar is explicitly linked to European regulatory frameworks. including the GDPR, AI Act, MDR, and EHDS regulation, ensuring that governance practices are not only technically robust but also legally grounded. For clarity, Table 5 summarises the proposed governance pillars, associated reporting requirements, and their alignment with key EU regulatory instruments. By embedding these dimensions into evaluation protocols and reporting standards, the SID-GT promotes transparency, accountability, and comparability across projects, facilitating trust and interoperability within EHDS.

In conclusion, when developed and governed responsibly, synthetic data can revolutionise medical research and healthcare delivery across Europe. The EHDS provides a promising foundation, but its success depends on concerted efforts to integrate ethics, transparency, and standardisation into every phase of synthetic data innovation. The introduction of a practical governance toolkit (Table 5) illustrates how these principles can be operationalised, providing a concrete pathway to ensure that synthetic imaging data are used in a manner that is legally robust, ethically sound, and clinically meaningful.
